# Age-related changes in lens thickness in children aged 3–17 years and its association with myopia and ocular biological parameters: a cross-sectional and longitudinal study

**DOI:** 10.3389/fped.2026.1807142

**Published:** 2026-05-15

**Authors:** Qing Yuan, Kexin Fan, Yan Lu

**Affiliations:** Department of Ophthalmology, Beijing Shijitan Hospital, Capital Medical University, Beijing, China

**Keywords:** anterior chamber depth, axial length, children, lens thickness, myopia, refractive development

## Abstract

**Objective:**

To investigate age-related changes in lens thickness(LT)and its associations with ocular biometrics and refractive status in children aged 3–17 years.

**Methods:**

A retrospective analysis of 699 children (1,398 eyes) undergoing ophthalmic examinations (June–August 2023).LT,axial length (AL), anterior chamber depth (ACD), mean corneal curvature (Km), and central corneal thickness (CCT) were measured via IOL Master 700. Longitudinal data were collected at 1-year (*n* = 285) and 2-year (*n* = 135) follow-ups. Multiple linear regression and repeated measures ANOVA were employed.

**Results:**

The median of LT was 3.42 mm (25th percentile, P25: 3.28; 75th percentile, P75: 3.56), which was significantly negatively correlated with age (r = −0.434, *P* < 0.001).LT exhibited a triphasic pattern:significant decrease before age 10(3.66→3.43→3.35 mm; *P* < 0.001), stabilization at 10–12 years,and mild thickening after age 13(+0.01 mm/year). Females demonstrated thicker LT than males (3.46 ± 0.22 vs.3.41 ± 0.20 mm; *P* < 0.001). LT negatively correlated with AL(r = −0.512) and ACD(r = −0.595), but not Km or CCT. Non-myopic children had thicker LT than myopic counterparts (3.54 ± 0.22 vs.3.35 ± 0.15 mm; *P* < 0.001). Longitudinally, LT decreased in ages 3–9,stabilized at 10–12, and increased in 13–17 years. Multiple regression identified ACD (*β*=−0.498), age (*β*=−0.121), and Km(*β*=−0.119) as independent LT predictors (R^2^ = 0.473).

**Conclusion:**

LT follows an age-dependent pattern of early decline, adolescent stabilization, and subsequent thickening. Thicker lenses in non-myopic children suggest lenticular compensation in refractive development. Age 10 represents a critical intervention window, with LT serving as a potential biomarker for pediatric refractive status assessment.

## 1 Introduction

Myopia has become one of the foremost global public health issues, with the global prevalence rate projected to rise to 49.8% by 2050, particularly alarming in children and adolescents (1, 2). The axial length (AL), corneal curvature (Km), and lens refractive power are the three core factors regulating refractive status in children (3). Corneal curvature tends to stabilize after the age of 2 (3), while lens refractive power gradually declines with age, maintaining emmetropic balance through compensatory myopia induced by axial length growth (4, 5). However, previous studies on pediatric refractive development have predominantly focused on the dynamic changes of AL and Km (6, 7), with relatively insufficient attention to lens morphology, particularly lens thickness (LT). As a key parameter reflecting lens structure and function, the age-related patterns of LT changes, influencing factors, and its association with myopia remain critical gaps in refractive development research. Existing studies yield conflicting conclusions: Han et al. (8) found that LT in children under 11 years of age significantly decreases with age, while increasing after 11 years; Wong et al. (9) reported a LT trough at 9 years in non-myopic children and at 10 years in myopic children; moreover, gender effects on LT and the direction of its association with myopia remain inconsistent across studies (4, 10–12). Additionally, most previous studies were cross-sectional designs, lacking large-sample, multi-timepoint longitudinal data to elucidate the stage-dependent characteristics of LT changes and their dynamic relationship with refractive status. Based on this, the present study adopted a mixed design of “cross-sectional + longitudinal follow-up”, enrolled 699 children aged 3–17 years, systematically analyzed the age-related changes in LT, explored the effects of gender, ocular biological parameters, and refractive status on LT, and validated the stability of LT changes through 1-year and 2-year longitudinal data. This provides high-quality clinical evidence for a deeper understanding of the emmetropization process in children and clarifying the compensatory mechanisms of myopia development, while also offering references for individualized formulation of myopia prevention and control strategies.

## 2 Materials and methods

### 2.1 Study subjects

This study was a retrospective study that enrolled 699 children aged 3–17 years (1,398 eyes) who visited the ophthalmology outpatient department of Beijing Shijitan Hospital, Capital Medical University, from June to August 2023.

Inclusion criteria: (1) Clear refractive media in both eyes (no corneal, lens, or vitreous opacities); (2) Corrected visual acuity ≥20/40 in children aged 3–4 years and ≥20/20 in children aged 5 years and above (meeting international pediatric visual screening standards); (3) Absence of ocular surface diseases (e.g., conjunctivitis, keratitis) and intraocular diseases (e.g., glaucoma, cataract, retinopathy, etc.); (4) No history of ocular surgery or trauma; (5) No history of contact lens wear (to avoid corneal morphological changes interfering with ocular biomarker measurements).

Exclusion criteria: (1) Refractive medium opacity affecting parameter measurement; (2) Corrected visual acuity not meeting the minimum standard for the corresponding age; (3) Concurrent ocular diseases or history of ocular surgery/trauma; (4) History of contact lens use; (5) Incomplete clinical data or lost to follow-up.

This study retrospectively enrolled all eligible children who visited the ophthalmology clinic during the study period (June–August 2023), no sampling method was used. Sample size was calculated for correlation analysis in cross-sectional studies: with reference to the correlation coefficient r = 0.5 between LT and AL, *α* = 0.05, power (1−*β*) = 0.90, the minimum required sample size was 102 subjects (204 eyes). We finally enrolled 699 subjects (1398 eyes), which was far larger than the minimum sample size, indicating that the study had adequate statistical power.

Follow-up response rate: 285 cases completed 1-year follow-up, with a response rate of 40.8%; 135 cases completed 2-year follow-up, with a response rate of 19.3%.

### 2.2 Research equipment and measurement methods

Ocular biometric measurements: The ZEISS IOL Master 700 biometer (Zeiss, Germany) was used by the same skilled technician under natural pupil conditions. Three measurements were taken per eye, with the mean value used as the final data. The measured parameters included: lens thickness (LT), axial length (AL), anterior chamber depth (ACD), mean corneal curvature (Km), and central corneal thickness (CCT).

Refraction examination: For myopic patients, the refraction examination was performed with the ciliary muscle paralyzed. Compound tropicamylamine eye drops were instilled into both eyes every 5 min for a total of 6 times. After 30 min of ocular rest, cycloplegic refraction was performed to determine the spherical error (S) and cylindrical error (C), which were then converted to equivalent spherical error (SE) using the formula SE = S + (C/2). The diagnostic criterion for myopia was SE ≤ −0.50D (1, 2).

Follow-up protocol: Prospective follow-up was conducted for eligible children. Among them, 285 cases (570 eyes) completed the 1-year follow-up examination, and 135 cases (270 eyes) completed the 2-year follow-up examination. All examinations were performed by the same technician using the identical ZEISS IOL Master 700 device to ensure consistent measurement conditions.

### 2.3 Ethical review

This study was conducted in strict compliance with the principles of the Declaration of Helsinki. As a retrospective study, an informed consent waiver was obtained for the ethical review.

### 2.4 Statistical methods

Data analysis was performed using SPSS 21.0 statistical software. The Shapiro–Wilk test was first applied to verify the distribution type of the measurement data. Since the LT data did not follow a normal distribution (*P* < 0.05), non-parametric tests were employed for intergroup comparisons: Kruskal–Wallis H test was used for LT comparisons between different age groups, with Bonferroni correction applied for pairwise comparisons; Mann–Whitney U test was used for LT comparisons between different genders, as well as between myopic and non-myopic groups.

Spearman correlation analysis was employed to assess the associations between age, AL, Km, ACD, CCT, and LT. The change in LT from baseline to 1 year was compared using paired-sample t-test; the changes from baseline to 1 and 2 years were analyzed with repeated-measures ANOVA; longitudinal changes in LT across different age groups were evaluated using two-way ANOVA.

Multiple linear regression analysis was employed to investigate the independent influencing factors of LT. The included variables were gender, age, AL, Km, ACD, and CCT. Forced inclusion method was adopted, and collinearity test was performed, with no significant collinearity observed. A *P*-value <0.05 was considered statistically significant.

## 3 Results

### 3.1 Distribution of baseline data

This study enrolled 699 children aged 3–17 years (1,398 eyes), including 359 males (51.4%) and 340 females (48.6%). Refractive status was classified as binocular myopia (373 cases, 53.4%), monocular myopia (36 cases, 5.2%), or non-myopia (290 cases, 41.5%). Since monocular myopia patients often exhibited one myopic eye and one non-myopic eye, direct classification into a single refractive group was impractical. As statistical analysis was performed per eye, baseline refractive status was categorized into three groups: binocular myopia, monocular myopia, and non-myopia. The gender distribution and refractive status distribution (number of individuals + number of eyes) across age groups are shown in Table 1, ensuring balanced sample composition in each subgroup to minimize confounding effects.

**Table 1:** Distribution of sex and refractive status by age group [*n* (%)].

age group (year)	Total number of cases (human being)	the male sex (human being)	Femininity (human being)	bilateral myopia Number of people/eyes	monocular myopia Number of people/eyes	Non-myopia Number of people/eyes
3–6	130	65（50.0）	65（50.0）	6/12	0/0	124/248
7–9	212	104（49.1）	108（50.9）	96/192	11/11	105/221
10–12	209	109（52.2）	100（47.8）	140/280	18/18	51/120
13–17	148	81（54.7）	67（45.3）	131/262	7/7	10/27
Total	699	359（51.4）	340（48.6）	373/746	36/36	290/616

Calculation logic for the number of eyes: Number of bilateral myopic eyes, number of bilateral myopia cases  ×   2; Number of monocular myopic eyes, number of monocular myopia cases  ×   1; Number of non-myopic eyes, number of non-myopia cases  ×   2 + number of monocular myopia cases  ×   1 (the fellow eye of monocular myopia was non-myopic).

### 3.2 Correlation between Lens thickness (LT) and age and comparison among age groups

The median LT in children across all age groups was 3.42 mm (P25: 3.28, P75: 3.56), and a significant negative correlation was found between LT and age (r = −0.434, *P* < 0.001). The distribution of LT across different age groups showed distinct stage-dependent characteristics with consistent trends in median values: the thickest LT was observed in the 3–6 years group (median: 3.65 mm, P25: 3.51, P75: 3.78), followed by gradual decreases in the 7–9 years group (median: 3.42 mm, P25: 3.32, P75: 3.52) and 10–12 years group (median: 3.34 mm, P25: 3.25, P75: 3.45). The 13–17 years group (median: 3.35 mm, P25: 3.27, P75: 3.44) showed a slight increase in LT compared with the 10–12 years group, with no significant between-group difference (*P* = 0.286).

**Figure 1:**
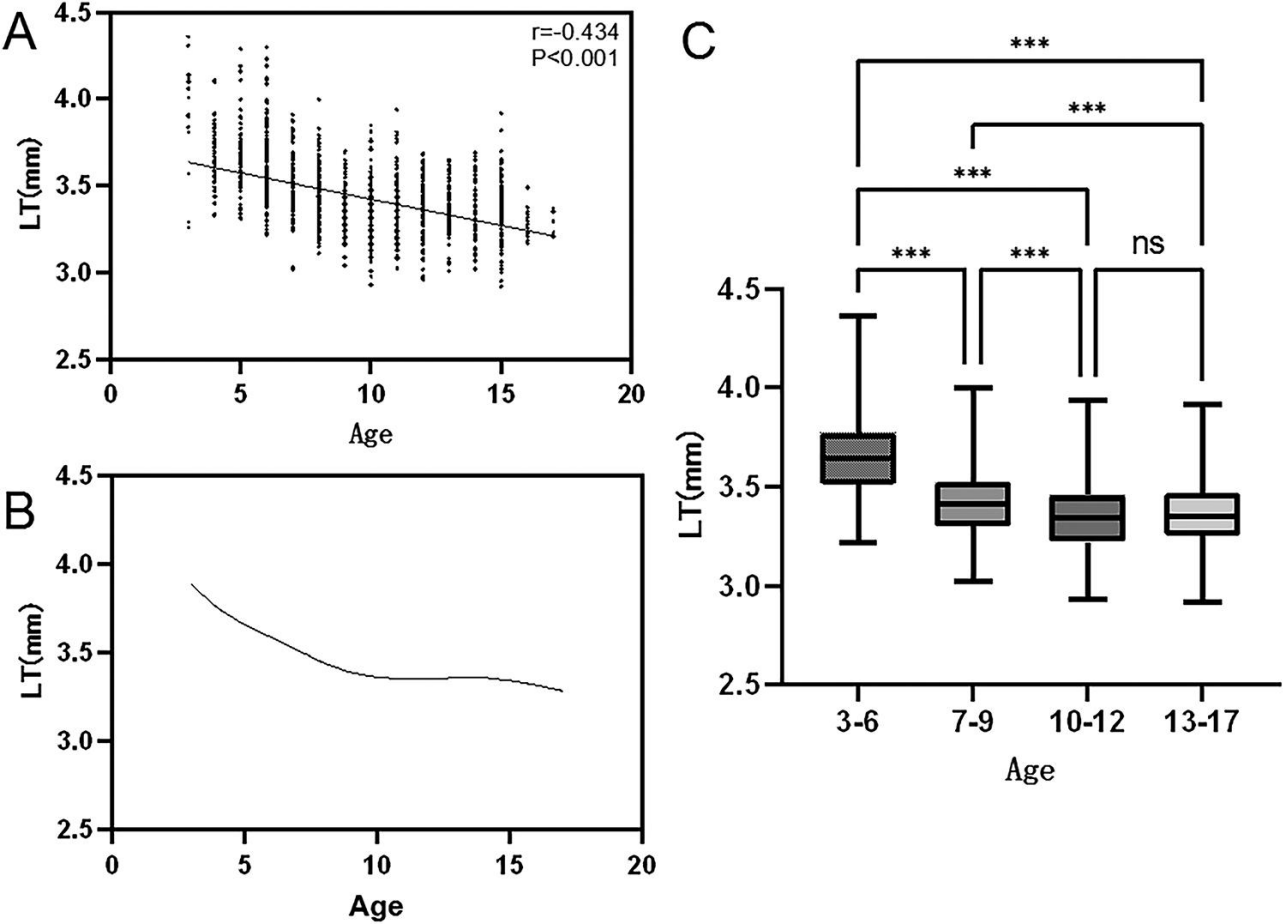
Comparison of LT across different age groups and correlation analysis between age and LT. (**A**) Correlation analysis between age and LT: Age was negatively correlated with LT, with a Spearman correlation coefficient of r = −0.434 (*P* < 0.001). (**B**) Comparison of LT across different age groups: The 3–6 years group showed significant differences compared with the 7–9, 10–12, and 13–17 years groups (*P* < 0.001); the 7–9 years group also showed significant differences compared with the 10–12 and 13–17 years groups (*P* < 0.001); no significant difference was found between the 10–12 and 13–17 years groups (*P* = 0.286, Kruskal–Wallis H test). (**C**) Changes in LT with age. The curve was generated by LOWESS fitting; median LT of all age groups was 3.42 mm (P25: 3.28, P75: 3.56). Estimated annual thinning before age 10 was 0.04 mm (95%CI: 0.03 to 0.05 mm), and estimated annual thickening after age 13 was 0.01 mm (95%CI: 0.00 to 0.02 mm).

Between-group comparisons revealed significant differences in LT between the 3–6 years group and all other age groups, as well as between the 7–9 years group and the 10–12 years, 13–17 years groups (all *P* < 0.001). LOWESS curve fitting indicated that approximately 10 years of age marked the inflection point in LT changes, with a rapid reduction before age 10 (estimated annual thinning: 0.04 mm, 95%CI: 0.03 to 0.05 mm), a slowed reduction from 10 to 12 years, and a transition to mild thickening after age 13 (estimated annual thickening: 0.01 mm, 95%CI: 0.00 to 0.02 mm) (Table 2, Figure 1).

**Table 2:** Comparison of lens thickness (LT) in children of different age groups (mm, $\bar{x}$ ±s).

Age Group (years)	Eyes (n)	Median (P25, P75) (mm)	*P*-value (intergroup comparison)
All ages	699 (1,398)	3.42 (3.28, 3.56)	<0.001
3–6	130 (260)	3.65 (3.51, 3.78)	–
7–9	212 (424)	3.42 (3.32, 3.52)	–
10–12	209 (418)	3.34 (3.25, 3.45)	–
13–17	148 (296)	3.35 (3.27, 3.44)	–

LT in the 3–6 years group was significantly different from that in the 7–9, 10–12, and 13–17 years groups (all *P* < 0.001). LT in the 7–9 years group was significantly different from that in the 10–12 and 13–17 years groups (all *P* < 0.001). No significant difference was found between the 10–12 and 13–17 years groups (*P* = 0.286; Kruskal–Wallis H test with Bonferroni correction).

### 3.3 Comparison of LT between children of different sexes

**Figure 2:**
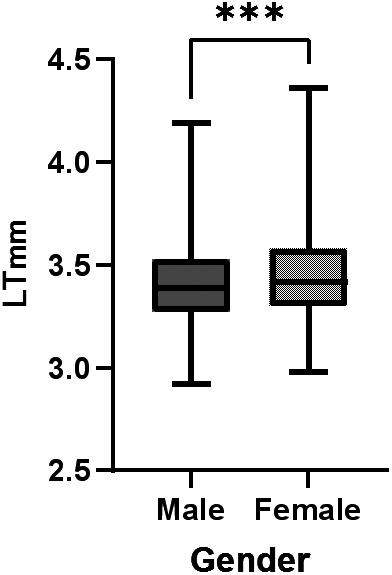
Comparison of LT between different gender groups. Median LT was 3.40 mm (P25: 3.26, P75: 3.53) in males and 3.45 mm (P25: 3.31, P75: 3.58) in females, with a median difference of 0.05 mm (95%CI: 0.03 to 0.07 mm), and the difference was statistically significant (*P* < 0.001, Mann–Whitney U test).

The median LT of male children was 3.40 mm (P25: 3.26, P75: 3.53), while that of female children was 3.45 mm (P25: 3.31, P75: 3.58). Mann–Whitney U test showed that LT was significantly thicker in females than in males, with a median difference of 0.05 mm (95%CI: 0.03 to 0.07 mm), and the difference was statistically significant (*P* < 0.001) (Figure 2).

### 3.4 Correlation between LT and ocular biological parameters

**Figure 3:**
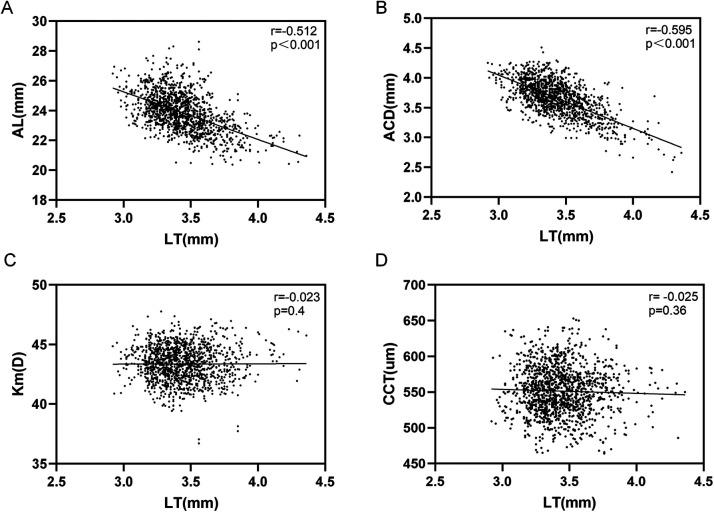
Correlation analysis (spearman correlation analysis) between different ocular biological measurement parameters and LT. (**A**) LT was negatively correlated with AL (r = −0.512, *P* < 0.001). (**B**) LT was negatively correlated with ACD (r = −0.595, *P* < 0.001). (**C**) No significant linear correlation was found between LT and Km (r = −0.023, *P* = 0.400). (**D**) No significant linear correlation was found between LT and CCT (r = −0.025, *P* = 0.360).

Spearman correlation analysis revealed distinct correlations between LT and ocular biological parameters:

A significant negative correlation with axial length (AL) (r = −0.512, *P* < 0.001);

A strong negative correlation with anterior chamber depth (ACD) (r = −0.595, *P* < 0.001);

No significant linear correlation with mean corneal curvature (Km) (r = −0.023, *P* = 0.400);

No significant linear correlation with central corneal thickness (CCT) (r = −0.025, *P* = 0.360) (Figure 3).

### 3.5 Impact of refractive Status on LT

**Figure 4:**
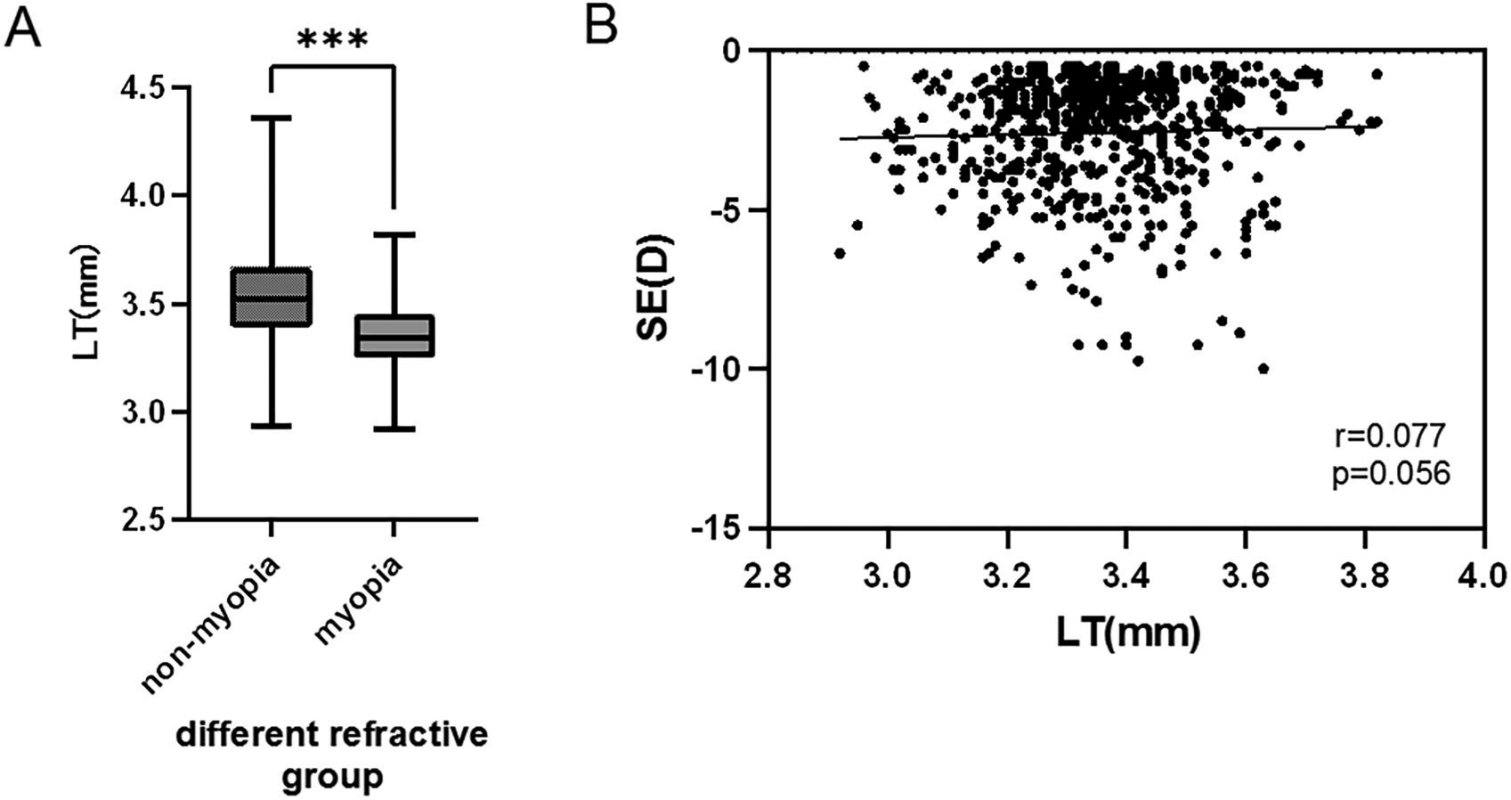
Comparison of refractive power with LT. (**A**) Comparison of LT between non-myopic and myopic groups: Median LT was 3.53 mm (P25: 3.39, P75: 3.68) in the non-myopic group and 3.34 mm (P25: 3.25, P75: 3.43) in the myopic group, with a median difference of 0.19 mm (95%CI: 0.17 to 0.21 mm), and the difference was statistically significant (*P* < 0.001, Mann–Whitney U test). (**B**) No significant linear correlation was found between refractive error and LT in the myopia group (r = 0.077, *P* = 0.056, Spearman correlation).

The median LT of the non-myopic group was 3.53 mm (P25: 3.39, P75: 3.68), while that of the myopic group was 3.34 mm (P25: 3.25, P75: 3.43). Mann–Whitney U test showed that LT was significantly thicker in the non-myopic group than in the myopic group, with a median difference of 0.19 mm (95%CI: 0.17 to 0.21 mm), and the difference was statistically significant (*P* < 0.001) (Figure 4A). Further analysis showed no significant linear correlation between refractive error and LT within the myopia group (r = 0.077, *P* = 0.056) (Figure 4B).

### 3.6 Independent factors of LT: multiple linear regression analysis

Gender, age, mean corneal curvature (Km), anterior chamber depth (ACD), and central corneal thickness (CCT) were included in the multiple linear regression model using the forced entry method. Variance inflation factor (VIF) testing showed that all variables had VIF < 3, indicating no significant multicollinearity, and the model satisfied the independence assumption.

**Figure 5:**
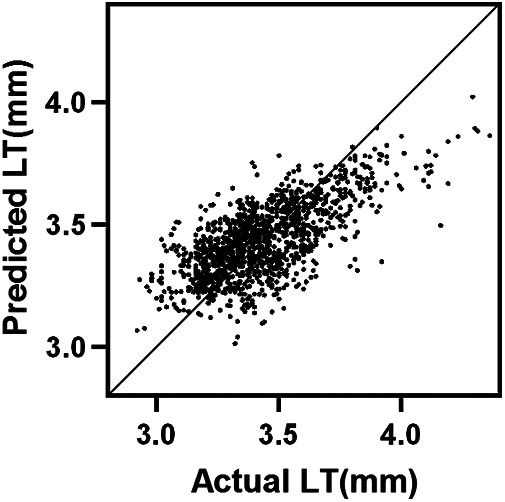
The multiple linear regression equation for LT. The regression equation for LT was: LT = 6.200−0.365 ×  ACD−0.017 ×  Km−0.011 ×  Gender (male=1, female=2)−0.008  ×  Age + 0.0002  ×  CCT. Model R2 = 0.473, *P* < 0.001.

The results demonstrated that ACD (*β*=−0.3645, 95%CI: −0.4031 to −0.3259, standardized *β*=−0.498, *P* < 0.001), age (*β*=−0.0079, 95%CI: −0.0116 to −0.0043, standardized *β*=−0.121, *P* < 0.001), and Km (*β*=−0.0173, 95%CI: −0.0243 to −0.0103, standardized *β*=−0.119, *P* < 0.001) were independent predictors of LT. The model fit was good with R^2^ = 0.473, suggesting that the included variables collectively explained 47.3% of the variation in LT. Gender (*β*=−0.0112, 95%CI: −0.0281 to 0.0056, standardized *β*=−0.027, *P* = 0.191) and CCT (*β*=0.0002, 95%CI: −0.00001 to 0.0005, standardized *β*=0.037, *P* = 0.064) showed no significant independent effect on LT (Table 3, Figure 5).

**Table 3:** Multivariate linear regression analysis of factors affecting Lens thickness (LT).

variable	Regression coefficient *β* (Estimate)	95%CI (profile likelihood)	standardization β	*P* price	VIF
nodal increment	6.200	5.709∼6.692	-	<0.001	-
Gender (Male=1, Female=2)	−0.0112	−0.0281∼0.0056	−0.027	0.191	1.104
Age (years)	−0.0079	−0.0116∼−0.0043	−0.121	<0.001	2.070
Mean corneal curvature (D)	−0.0173	−0.0243∼−0.0103	−0.119	<0.001	1.600
ACD （mm）	−0.3645	−0.4031∼−0.3259	−0.498	<0.001	1.904
Central corneal thickness (*μ*m)	0.0002	−0.00001∼0.0005	0.037	0.064	1.078
model goodness of fit	R^2^ = 0.4729, F = 208.018, *P* < 0.001	-	-	-	-

β, regression coefficient; 95%CI, 95% confidence interval; VIF, variance inflation factor; ACD, anterior chamber depth; CCT, central corneal thickness. The model showed good goodness of fit: R^2^ = 0.473, F = 208.018, *P* < 0.001. No significant multicollinearity was detected (all VIF < 3).

### 3.7 Longitudinal trend of LT

#### 3.7.1 Longitudinal changes across all age groups

Among 285 children who completed 1-year follow-up, baseline median LT was 3.45 mm (P25: 3.31, P75: 3.59), which decreased to 3.42 mm (P25: 3.28, P75: 3.56) at 1 year, with an estimated annual thinning of 0.03 mm (95%CI: 0.02 to 0.04 mm), and the difference was statistically significant (*P* < 0.001).

**Figure 6:**
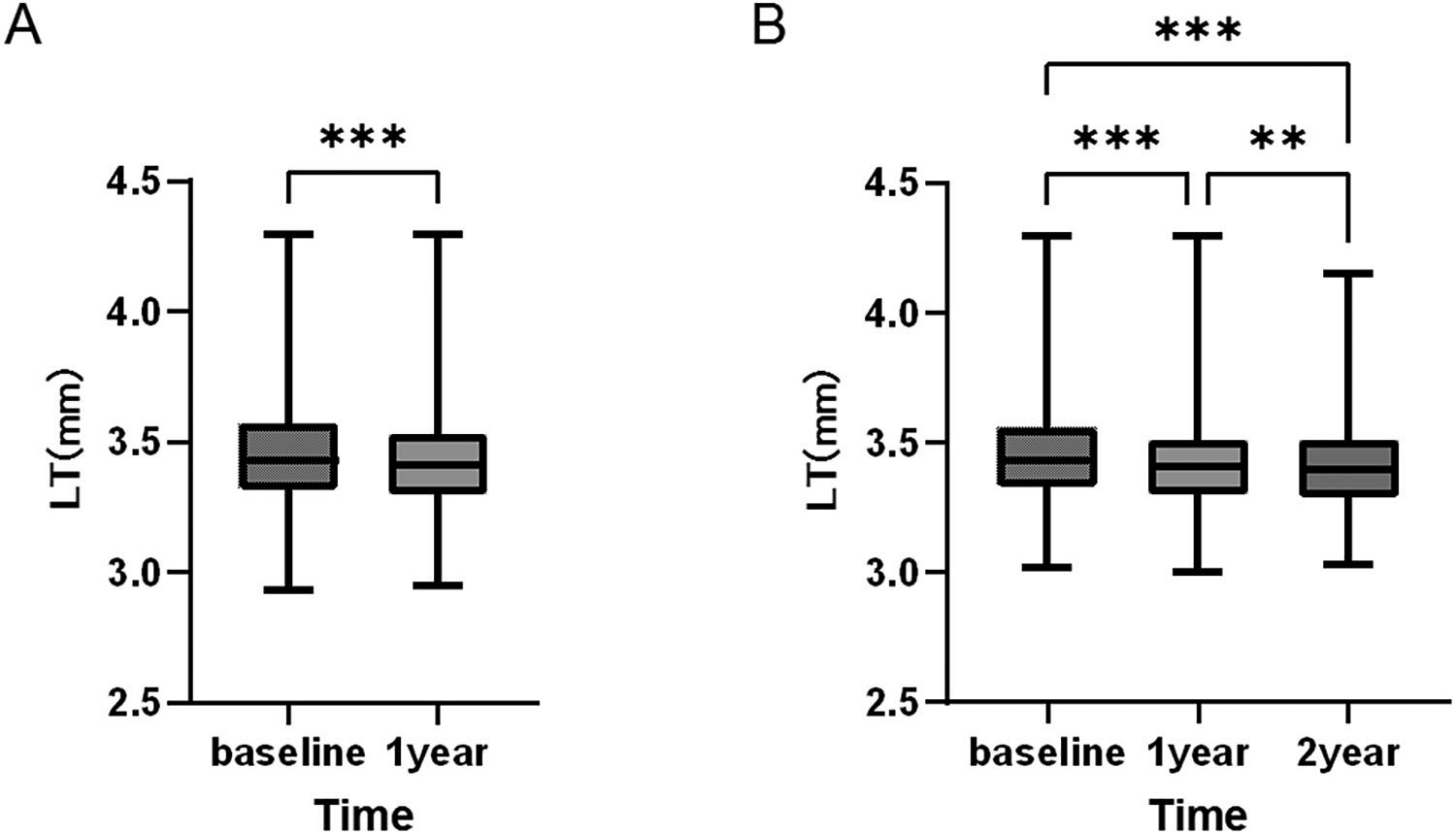
Longitudinal trend of lens thickness (LT) in children across all age groups. (**A**) One-year follow-up cohort: LT was significantly lower at 1 year than at baseline (*P* < 0.001), with an estimated annual thinning of 0.03 mm (95%CI: 0.02 to 0.04 mm). (**B**) Two-year follow-up cohort: Significant differences were found in LT among baseline, 1 year, and 2 years (*P* < 0.001), with a cumulative 2-year thinning of 0.05 mm (95%CI: 0.03 to 0.07 mm).

Among 135 children who completed 2-year follow-up, LT showed a continuous downward trend: baseline median LT was 3.46 mm (P25: 3.32, P75: 3.60), 3.42 mm (P25: 3.28, P75: 3.56) at 1 year, and 3.41 mm (P25: 3.27, P75: 3.55) at 2 years. Repeated-measures ANOVA showed significant differences in LT across follow-up time points (*P* < 0.001), and the sphericity assumption was met (*P* = 0.126). Bonferroni *post-hoc* tests revealed significant differences between baseline and 1 year, baseline and 2 years, and 1 year and 2 years (all *P* < 0.05), indicating a gradual reduction in LT with prolonged follow-up in children aged 3–17 years (Figure 6).

#### 3.7.2 Longitudinal changes stratified by age

**Figure 7:**
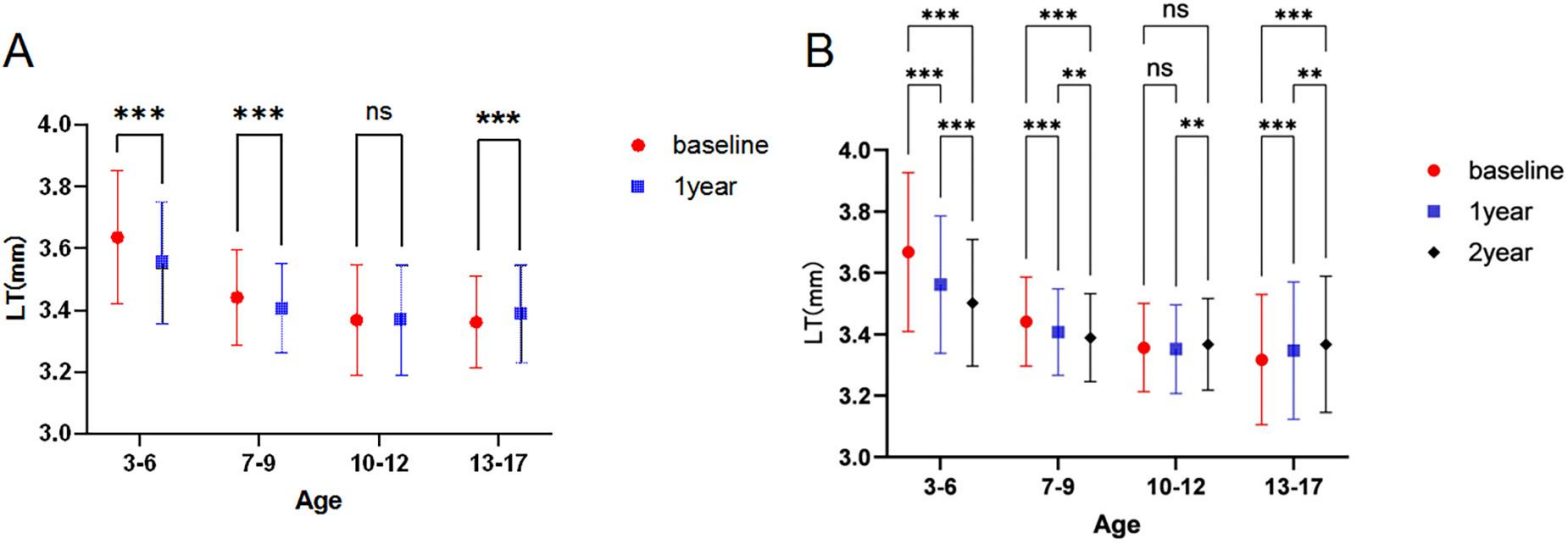
Changes in LT at different measurement times across age groups. (**A**) LT was significantly lower at 1 year than at baseline in the 3–6, 7–9, years groups (*P* < 0.001); no significant difference was found between 1-year and baseline LT in the 10–12 years group (*P* = 0.887). (**B**) In the 3–6 years group, all pairwise comparisons of LT among time points were significant (*P* < 0.001); in the 7–9 years group, significant differences were found between baseline and 1 year, baseline and 2 years (*P* < 0.001); in the 10–12 years group, a significant difference was found only between 1 and 2 years (*P* = 0.004); in the 13–17 years group, all pairwise comparisons were significant (*P* < 0.05) (two-way ANOVA).

Two-way ANOVA was performed with age and time as main effects and age   ×   time as the interaction effect to analyze age-stratified longitudinal changes in LT. The results showed a significant interaction effect between age and time on LT (*P* < 0.001), and the main effects of age and time were also statistically significant, with distinct longitudinal trends across age groups (Figure 7, Table 4).

**Table 4:** Two-way ANOVA results for the interaction of age and time on lens thickness (LT).

Source of variation	Sum of Squares (SS)	Degrees of Freedom (df)	F value	*P* value
Age	8.254	3	68.78	<0.001
Time	1.026	2	25.65	<0.001
Age × Time	3.482	6	42.36	<0.001
Error	5.973	268	-	-
Total	18.735	279	-	-

Statistical method: two-way analysis of variance (ANOVA); age and time were main effects, and age  ×   time was the interaction effect. *P* < 0.05 was considered statistically significant.

3–6 years group: In the 1-year follow-up cohort, estimated annual LT thinning was 0.09 mm (95%CI: 0.07 to 0.11 mm); in the 2-year follow-up cohort, cumulative 2-year LT thinning was 0.17 mm (95%CI: 0.15 to 0.19 mm), with significant differences at all time points (all *P* < 0.001).

7–9 years group: In the 1-year follow-up cohort, estimated annual LT thinning was 0.03 mm (95%CI: 0.02 to 0.04 mm); in the 2-year follow-up cohort, cumulative 2-year LT thinning was 0.05 mm (95%CI: 0.03 to 0.07 mm), with significant differences at all time points (all *P* < 0.05).

10–12 years group: No significant change in LT was found in the 1-year follow-up cohort (*P* = 0.887); in the 2-year follow-up cohort, cumulative 2-year LT thickening was 0.01 mm (95%CI: 0.00 to 0.02 mm), with a significant difference only between 1 and 2 years (*P* = 0.004).

13–17 years group: In the 1-year follow-up cohort, estimated annual LT thickening was 0.03 mm (95%CI: 0.01 to 0.05 mm); in the 2-year follow-up cohort, cumulative 2-year LT thickening was 0.09 mm (95%CI: 0.07 to 0.11 mm), with significant differences at all time points (all *P* < 0.05).

### 3.8 Synergistic longitudinal changes of axial length (AL) and LT

In the same 2-year follow-up cohort, AL and LT showed opposite longitudinal trends:

In the 3–9 years group, AL continued to increase with an estimated annual increase of 0.32 mm (95%CI: 0.28 to 0.36 mm), while LT continued to decrease with an estimated annual thinning of 0.042 mm (95%CI: 0.031 to 0.053 mm).

In the 13–17 years group, AL growth slowed with an estimated annual increase of 0.15 mm (95%CI: 0.10 to 0.20 mm), while LT shifted to thickening with an estimated annual increase of 0.011 mm (95%CI: 0.007 to 0.015 mm).

These synergistic changes further support the mechanism that LT compensates for AL growth through thickness adjustment (Table 5).

**Table 5:** Synergistic longitudinal changes of axial length (AL) and lens $\bar{x}$ thickness (LT) (mm, $\bar{x}$ ±s).

Age Group (years)	Parameter	Baseline (mm)	1-year Follow-up (mm)	2-year Follow-up (mm)	1-year Change (mm)	95% CI	2-year Change(mm)	95% CI	*P* (1y vs. Baseline)	*P* (2y vs. Baseline)	*P* (2y vs. 1y)
3–6	AL	22.15 ± 0.68	22.47 ± 0.70	22.78 ± 0.72	0.32	0.28–0.36	0.63	0.57–0.69	<0.001	<0.001	<0.001
	LT	3.67 ± 0.21	3.61 ± 0.19	3.55 ± 0.17	–0.06	–0.08 to –0.04	–0.12	–0.15 to –0.09	<0.001	<0.001	<0.001
7–9	AL	23.24 ± 0.75	23.56 ± 0.76	23.85 ± 0.78	0.32	0.29–0.35	0.61	0.56–0.66	<0.001	<0.001	0.006
	LT	3.44 ± 0.17	3.40 ± 0.16	3.37 ± 0.15	–0.04	–0.06 to –0.02	–0.07	–0.09 to –0.05	<0.001	<0.001	0.002
10–12	AL	24.12 ± 0.82	24.30 ± 0.83	24.45 ± 0.84	0.18	0.14–0.22	0.33	0.28–0.38	<0.001	<0.001	<0.001
	LT	3.35 ± 0.17	3.34 ± 0.16	3.36 ± 0.15	–0.01	–0.03 to 0.01	0.01	–0.01 to 0.03	0.887	0.150	0.010
13–17	AL	24.85 ± 0.90	24.98 ± 0.91	25.08 ± 0.92	0.13	0.08–0.18	0.23	0.17–0.29	<0.001	<0.001	0.003
	LT	3.35 ± 0.16	3.38 ± 0.15	3.41 ± 0.14	0.03	0.01–0.05	0.06	0.04–0.08	<0.001	<0.001	0.008

1. AL, axial length; LT, lens thickness. 2. Change, follow-up value−baseline value; positive change for AL indicates axial elongation; negative change for LT indicates lens thinning; positive change for LT indicates lens thickening. 3. All changes were reported with estimated effect size and 95%CI. 4. Statistical method: repeated-measures ANOVA; *P* < 0.05 was considered statistically significant.

## 4 Discussion

This study, utilizing large-sample cross-sectional and two-year longitudinal follow-up data, confirms that the lens thickness (LT) in children aged 3–17 years follows an age-dependent pattern of “rapid decline before 10 years, stabilization between 10 and 12 years, and mild thickening in the 13–17 years old group”. This finding aligns with the results of Han et al. (8) (inflection point at 11 years) and Wong et al. (9) (inflection point at 9–10 years), further validating the U-shaped curve characteristic of childhood LT changes. The study also quantified the rate of change at each stage (an average annual decrease of 0.04 mm before 10 years and an average annual increase of 0.01 mm after 13 years), providing more precise data support for establishing biological reference standards for pediatric refractive development.

Three primary hypotheses currently exist regarding the mechanism of lens thickness (LT) changes: 1) The mechanical tension theory (12, 13), which posits that rapid axial elongation exerts a tensile force on the equatorial plane of the lens, leading to its thinning. After age 10, axial elongation slows, the tensile force diminishes, and lens thickness increases; 2) The lens cortex-nucleus balance theory (5), suggesting that pre−10-year-old lens nucleus compression exceeds cortical fiber growth, resulting in LT reduction. After age 10, nucleus compression moderates, and cortical fiber growth predominates, leading to LT thickening; 3) The emmetropization compensation theory (8), which proposes that the lens compensates for axial elongation-induced myopia through thinning to maintain refractive balance. The newly added longitudinal data on the coordinated changes of axial length (AL) and LT in this study demonstrate that in the 3–9 age group, rapid AL growth (0.32 mm/year) coincides with rapid LT reduction (0.042 mm/year), while in the 13–17 age group, AL growth slows (0.15 mm/year) synchronizes with LT thickening. These findings support the synergistic effects of the “emmetropization compensation theory” and “mechanical tension theory” —the lens undergoes passive stretching (mechanical tension) and active morphological adjustment (compensation mechanism) to thin during early childhood, while post-adolescence axial elongation decelerates, highlighting the lens's intrinsic growth potential and gradual thickness increase.

Multiple linear regression analysis revealed that ACD was the most significant independent factor influencing LT (standardized *β*=−0.498), followed by age (*β*=−0.121) and Km (*β*=−0.119), consistent with the findings of Lu et al. (10) and Wong et al. (9). The strong negative correlation between ACD and LT can be explained by ocular anatomical structures: limited anterior chamber space leads to reduced compression on the anterior lens surface when ACD increases, resulting in a natural decrease in thickness; conversely, when ACD is shallow, the lens must thicken to maintain refractive function (5). This study further demonstrated that the negative correlation between ACD and LT remains stable across all age groups, suggesting that the anatomical synergy between the anterior chamber and lens is an inherent characteristic throughout childhood. This finding provides a reference for refractive assessment in children with congenital anterior chamber developmental abnormalities.

The negative correlation between axial length (AL) and lens thickness (LT) was more pronounced in the 3–9 age group (r = −0.536) and lessened in the 10–17 age group (r = −0.428). This difference may be attributed to variations in axial length growth rates: rapid axial elongation during early childhood exerts a more significant stretching effect on the lens, whereas the slowing growth after adolescence reduces this mechanical influence (9). The independent effect of corneal curvature (Km) on LT was weak (*β*=−0.119), and Spearman correlation analysis revealed no significant association between the two, suggesting that corneal curvature has limited direct impact on lens thickness. Instead, it may indirectly regulate ocular refractive balance through indirect mechanisms, consistent with the established notion that “corneal curvature stabilizes after 2 years of age” (3).

This study found that the lens thickness (LT) in female children was significantly thicker than in males, and this difference was observed in all age groups except the 13–17 year-old group, supporting Warrier et al.'s (14) “refractive compensation hypothesis” —that the female axial length is typically shorter than the male, and the lens thickens to compensate for refractive insufficiency, thereby maintaining a myopic state. The disappearance of gender differences in the 13–17 age group may be related to accelerated axial length growth in adolescent females and the convergence of lens thickening magnitude with males. However, Li et al. (15) and Garcia-Domene et al. (16) did not find gender differences in LT, suggesting that the reasons for the discrepancy may lie in the sample age range (e.g., some studies excluded younger children aged 3–6 years), ethnic differences (Asian children vs. European and American children), and variations in measurement device precision. Further validation is required through multicenter studies with larger sample sizes.

Regarding the association between lens thickness (LT) and myopia, this study confirmed that non-myopic children exhibited significantly thicker LT compared to myopic children, with this difference observed across all age groups. These findings align with the conclusions of Shih et al. (11) and Praveen et al. (17), but contradict the conclusion by Mutti et al. (12) that “myopic children have thicker LT.” This discrepancy may stem from differences in study design: Mutti et al. (12) adjusted for vitreous cavity depth to exclude the influence of axial length differences, whereas our study did not perform this adjustment and included monocular myopic children (5.15%), which may have introduced bias. Additionally, Mutti et al. (12) studied newly developed myopia, while our study included children with long-term myopia, and the different stages of dynamic LT changes may have influenced the results. Our study found no significant correlation between refractive error and LT within the myopia group (r = 0.077, *P* = 0.056), suggesting that LT may be associated with the onset of myopia but not its progression. This supports the hypothesis that “lens morphological changes are an early compensatory mechanism for myopia rather than a driver of progression” (8).

This study has the following limitations: 1) The retrospective design led to selection bias, with the prevalence of myopia among enrolled outpatients (64.2%) being higher than the average prevalence (approximately 50%) among children aged 3–17 in Beijing (24), which may overestimate the association strength between LT and myopia; 2) The single-center sample (Beijing urban area) lacks data from rural and other regions, limiting extrapolation, and excludes children from ethnic minorities, resulting in insufficient racial representation; 3) Environmental confounding factors such as parental myopia history, daily outdoor activity time, and near work duration were not included, as these factors may indirectly modulate LT by affecting axial length growth; 4) The follow-up period was only 2 years, making it impossible to observe long-term evolution of LT after age 17, and functional parameters such as lens refractive power were not recorded, hindering a comprehensive assessment of the optical role of the lens; 5) The low 1-year (40.8%) and 2-year (19.3%) follow-up response rates may introduce selection bias in the longitudinal analysis, reduce the representativeness of the longitudinal dataset, and compromise the accuracy and generalizability of the findings regarding longitudinal changes in lens thickness.

Future research may focus on the following directions: 1) Multi-center, prospective cohort studies involving children from diverse geographical and ethnic backgrounds, integrating environmental and genetic factors to establish predictive models for LT changes; 2) Extended follow-up through early adulthood to clarify long-term trends of LT thickening and its association with high myopia complications (e.g., posterior scleral staphyloma); 3) Combined optical coherence tomography (OCT) measurements of lens cortex and nucleus thickness to investigate the structural basis of LT changes; 4) Exploration of LT as a biomarker threshold for myopia prediction, providing evidence for individualized myopia prevention and control strategies.

## 5 Conclusion

The lens thickness in children aged 3–17 years exhibits a distinct age-dependent pattern: rapid decline before 10 years of age, plateauing between 10 and 12 years, and mild thickening after 13 years. Anterior chamber depth (ACD) is the most prominent ocular biometric parameter correlated with lens thickness (LT) in multiple linear regression analysis, followed by axial length (AL), age, and mean corneal curvature (Km); these parameters jointly correlate with the morphological development of the lens in children with obvious anatomic synergy. Female children demonstrate significantly thicker lenses than males, and non-myopic children exhibit significantly thicker lenses than myopic children. These differences suggest that lens morphological changes may contribute to the pathogenesis of myopia through refractive compensation mechanisms. This study identifies 10 years as a critical inflection point in the lens thickness changes of children, which can serve as a key intervention window for refractive development monitoring and myopia prevention. It also confirms that lens thickness may become a potential biomarker for assessing refractive development status in children, providing crucial data support for early screening, risk warning, and individualized management of refractive errors in children.

## Data Availability

The original contributions presented in the study are included in the article/Supplementary Material, further inquiries can be directed to the corresponding author.
